# Hematological Malignancy-Derived Small Extracellular Vesicles and Tumor Microenvironment: The Art of Turning Foes into Friends

**DOI:** 10.3390/cells8050511

**Published:** 2019-05-27

**Authors:** Ernesto Gargiulo, Jerome Paggetti, Etienne Moussay

**Affiliations:** Tumor-Stroma Interactions, Department of Oncology, Luxembourg Institute of Health, 84, val fleuri, L-1526 Luxembourg, Luxembourg; ernesto.gargiulo@lih.lu

**Keywords:** tumor-derived small EVs, exosomes, tumor microenvironment, hematological malignancies, leukemia, lymphoma, myeloma, immunity

## Abstract

Small extracellular vesicles (small EVs) are commonly released by all cells, and are found in all body fluids. They are implicated in cell to cell short- and long-distance communication through the transfer of genetic material and proteins, as well as interactions between target cell membrane receptors and ligands anchored on small EV membrane. Beyond their canonical functions in healthy tissues, small EVs are strategically used by tumors to communicate with the cellular microenvironment and to establish a proper niche which would ultimately allow cancer cell proliferation, escape from the immune surveillance, and metastasis formation. In this review, we highlight the effects of hematological malignancy-derived small EVs on immune and stromal cells in the tumor microenvironment.

## 1. Introduction

Our review follows the “Minimal information for studies of extracellular vesicles 2018 (MISEV2018)” updated guidelines [1]. In order to guarantee correct divulgation and to increase the experiment reproducibility, vesicles nomenclature, characteristics and cited papers have been selected to follow the recommendations from the International Society for Extracellular Vesicles (ISEV) as closely as possible.

Small extracellular vesicles (small EVs), sometimes widely called exosomes, are vesicles (30–150 nm) released by all cells present in all body fluids that are involved in short- and long-distance cell communication [2,3,4,5,6]. Small EVs are typically included in the category of extracellular vesicles (EVs), together with exosome-like vesicles (20–50 nm), membrane particles (50–80 nm), microvesicles (100–1000 nm) and apoptotic bodies (<5000 nm) [1,7]. Beside the size, what distinguishes small EVs from other extracellular vesicles is their specific biogenesis, which is strictly bound to the endosomal compartment [1,8,9].

The release of small EVs is dependent on the cell of origin, the physiological context, and the purpose [8,10]. Initially considered a physiological phenomenon to remove cellular waste, today, small EVs are considered to be a sophisticated way for cells to communicate with the surrounding microenvironment [7,11]. Their molecular content is strictly dependent on the cell of origin [10]. Interestingly, it was shown that tumor-derived small EVs (TEVs) carry a cargo of molecules that is different from the small EVs released by the healthy counterpart [10]. Therefore, it is not surprising that tumor cells release specific small EVs containing an unique molecular fingerprint which is used by tumors to increase proliferation [12], metastasis formation [12,13] and immune escape [14], effectively reshaping the healthy microenvironment in favor of a pro-tumorigenic one.

## 2. Generality on Small EVs and the Tumor Microenvironment

### 2.1. Architecture and Content

Small EVs are vesicles delimited by a lipid bilayer membrane containing an impressive range of molecules such as nucleic acids (DNA and functionally active RNAs), proteins, lipids and metabolites [15,16,17,18]. It is important to mention that the presence of DNA inside small EVs is still a matter of debate in light of the recent reassessment of EV composition [19]. Although no unique marker has been identified, it is commonly recognized that the enrichment in specific molecules correlates to the small EV biogenesis. Indeed, small EVs contain endosome-associated proteins (Rab GTPase, SNAREs, Annexins, and flotillin), multivesicular endosome (MVE) proteins (Alix and Tsg101) and tetraspanins (CD63, CD81 and in minor amount CD9) [20,21,22]. The content listed in the following paragraph is not present in all small EVs but greatly varies depending on tissue/organ of origin and condition (healthy vs diseased).

Small EVs contain messenger RNAs that have been reported to modulate the cell cycle, migration and angiogenesis of target cells [23,24]. Furthermore, small EVs also contain selected microRNAs (miRNA) that are involved in the regulation of various cellular events, such as cell cycle, senescence, repair of DNA damage and apoptosis [25]. Concerning miRNAs, they can be divided into two categories in cancer: oncogenic tumor-inducer miRNAs (oncomiRs) and the tumor-suppressor miRNAs [26]. Depending on the category of the miRNAs, they may or may not favor the promotion of cancer growth and progression. A deep analysis of small EVs showed the presence of miRNAs typically involved in tumorigenesis (miR-17, miR-18, miR-19a, miR-19b-1, miR-20, and miR-93-1) and metastasis [27,28]. The mechanisms responsible for miRNAs sorting into small EVs are still under investigation. Several reports have suggested the presence of motifs controlling miRNAs loading into small EVs and the prominent role of sumoylated ribonucleoproteins [29] and of the proto-oncogene KRAS [30].

The characterization of lipids in small EVs is a more complex process to be assessed. The difficulty stands in the preparation of the small EVs and in the purity of the samples. Indeed lipoparticles and/or lipid droplets can easily be co-isolated with small EVs, resulting in inaccurate measurements. Small EVs predominantly contain lipids, such as cholesterol, diglycerides, sphingolipids, phospholipids and glycerophospholipids [31,32]. The ratio for certain small EV lipids is increased, compared to the cell of origin. These include sphingomyelin (SM), phosphatidylserine (PS), phosphatidylcholine (PC), phosphatidylinositol (PI) and cholesterol [32,33,34].

These lipids can be enriched up to four times in the composition of small EV membrane compared to cell membrane and play an important role in membrane rigidity of the vesicles [35,36]. This is in contrast with another study, in which similar lipid contents were reported in small EVs and corresponding parental cells [37]. Variations can therefore be observed according to the parent cell type of origin. Finally, small EVs have also been reported to contain bioactive lipids, such as prostaglandins (PGs) and leukotrienes, and activated enzymes involved in lipid metabolism [25,38,39].

In addition to lipids, small EVs carry a wide range of metabolites, such as phosphatidylglycerol, *N*-arachidonoyl-l-serine, sphingomyelin, coenzyme Q10 and malonyl CoA [40]. These metabolites have previously been described to be involved in cell proliferation, migration and angiogenesis as well as in cancer and aging [41,42]. Altadill and colleagues have shown that small EVs can reprogram the cell metabolic machinery upon uptake by cancer cells, suggesting that metabolite profiling can be used for differentiating cellular states [40]. Finally, another study demonstrated that small EVs from patient-derived cancer-associated fibroblasts (CAFs) reprogram the cellular machinery in cancer cells. In addition, they showed that the uptake of CAF-derived small EVs inhibited mitochondrial oxidative phosphorylation, but at the same time, that it increased glycolysis and glutamine-dependent reductive carboxylation [43].

### 2.2. The Complex Interactions between TEVs and Cells in the Microenvironment

Different studies have described the ability of TEVs to re-educate the surrounding cells in order to gain strategic advantages. Virtually all cell types were reported to be targeted by small EVs. Macrophages targeted by TEVs polarized to a more pro-tumorigenic phenotype (M2), in turn macrophage-derived small EVs stimulate invasion of cancer cells by transferring the protein Wnt5a [44]. In order to maintain a constant macrophage polarization, it is fundamental that TEVs regulate monocyte recruitment, survival and differentiation. TEVs improve monocyte survival through mitogen-activated protein kinase (MAPK) and receptor tyrosine kinases (RTK), such as epidermal growth factor receptor (EGFR) and human epidermal growth factor receptor 2 (HER2)-dependent mechanisms [45]. Immune cells are also strongly impacted by TEVs. CD8^+^ T lymphocytes circulating in cancer patients were found to highly express CD95 and programmed cell death protein 1 (PD-1) markers [46,47]. On the other hand, several other studies showed that cancer cells express the programmed death ligand 1 (PD-L1) and CD95 ligand (FasL) together with the corrupted microenvironment immune cells [48,49,50,51,52,53]. TEVs also carry FasL as well as PD-L1, and this association has recently linked TEVs with ligand-mediated apoptosis and exhaustion in T cells [54,55,56]. Szajnik et al. suggested that increased levels of phospho-STAT3, phospho-SMAD2/3, IL-10 and TGF-β1 expression in TEV-treated CD4^+^CD25^hi^FOXP3^+^ regulatory T cells (Tregs) may account for the reduced anti-tumor immune response in cancer patients [57]. In addition, TEVs can target and modify stromal and stem cells in the bone marrow. TEVs carrying specific RNAs activate Toll-like receptor 3 (TLR3) in stromal cells thus promoting tumor growth and establishing a proper tumor niche by inducing neutrophil recruitment and immobilization at the tumor location [58,59,60,61]. Hornick and colleagues also demonstrated that TEVs possess even a systemic effect. Indeed, Acute Myeloid Leukemia (AML)-derived EVs enriched in miR-150 and miR-155 impaired hematopoietic stem and progenitor cell (HSPC) clonogenicity through suppression of c-MYB translation [62].

TEVs are part of a complex strategy used by the tumor cells, not only to circumvent the canonical cellular defenses, but also to deeply re-educate immune and stromal cells towards a pro-tumorigenic phenotype.

### 2.3. TEVs as Potential Biomarkers

All these unique features have the potential to be used to discriminate small EVs released by normal cells from TEVs, potentially allowing a rapid detection of tumor development during cancer diagnosis and patients’ follow-up [10,63,64,65,66]. Indeed, relapse is still the main cause of mortality in treated cancer patients. Establishing new methods to track minimal residual disease (MRD) through detection of TEVs could represent an innovative strategy to guarantee an optimal patient quality of life with better adjusted treatments and minimized relapse occurrence.

Today, proteins and miRNAs are the most commonly used biomarkers. It was recently shown that pancreatic cancer patients present circulating TEVs highly enriched in glypican-1 (GPC1) compared with healthy controls, this marker has been described to specifically correlate with early stage of pancreatic cancer [67]. Hepatoma-derived small EVs exhibit high level of miR-103 which is transferred to endothelial cells inducing metastasis formation, and was therefore suggested as predictive marker [68]. In line with this, macrophage migration inhibitory factor (MIF) is detectable in TEVs of stage I pancreatic patients with high potential for metastasis in the liver [69].

Recently, Manier and colleagues linked the reduction of plasma small EV miRNA let-7b and miR-18a with multiple myeloma (MM) patient poor survival [70]. This confirmed the already known importance of these miRNAs in cancer progression and suggested their significant correlation with negative progression-free survival and overall survival [70,71,72]. Similarly a combined detection of miRNA-150, -155, and -1246 in AML-derived small EVs was proposed as marker to monitor patients following treatment [73]. Furthermore, the cargo protein TGF-β1 has also been suggested as potential biomarker for AML patients subjected to post-chemotherapy consolidation supportive therapy [74,75]. Similarly, a decreased level of vimentin in TEVs after gemcitabine treatment suggested it as a potential biomarker for pancreatic cancer [76].

Despite several studies that have suggested TEVs as potential biomarkers, it is important to pay attention to the protocols used for isolation, quality control and characterization [1,77,78]. Indeed, due to their small size, heterogeneity, the presence of contaminants (e.g., soluble proteins) and isolation methods used, small EV detection and correct classification can be challenging, and thus, caution must be applied. Consequently, it is important to consider these issues when making definitive conclusion about small EV cargo, function and characterization [1,79].

## 3. Hematological Malignancy-Derived Small EVs and Immune Cells

Of the wide ranging of studies describing the role of TEVs in tumorigenesis, in this review, we will focus our attention on hematological malignancy-derived small EVs and their impact on immune and stromal cells of the tumor microenvironment (TME). 

**Figure 1:**
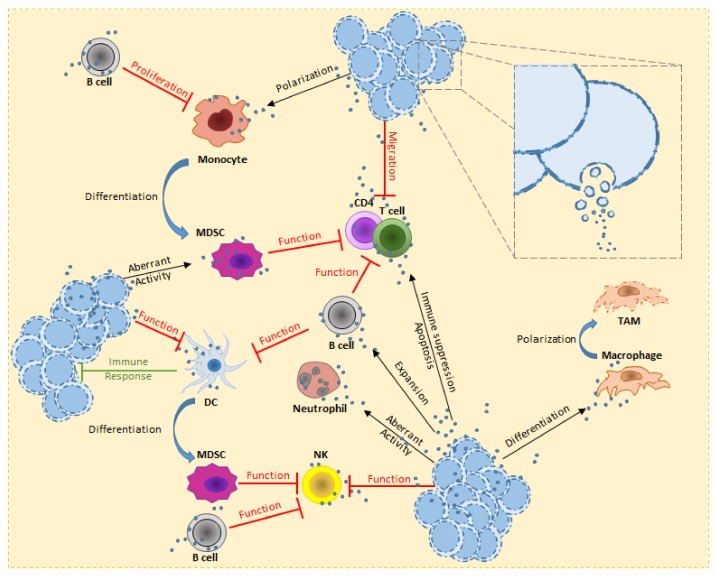
Overview of hematological malignancy-derived small EV effects on immune cells. In order to escape from the immune system, tumor cells deploy several strategies. TEVs are used to stimulate B cell expansion and activity against several immune cells (e.g., T cells and NK). T cells are known to be affected on the proliferative potential and cytokine production. TEVs directly reduce the T cells migration, leading to up-regulation of receptors involved in immune suppression and or activate pro-apoptotic signals. Alternatively, MDSCs acquire strong immune suppression activity, either from interaction of TEVs or aberrant differentiation from myeloid cells (e.g., monocytes) or immature DCs. Under the effect of TEVs, monocytes polarize towards a pro-tumorigenic form. This leads to pro-inflammatory cytokines release and expression of receptors involved in immune suppression. NK cells have high cytotoxic activity, the tumor uses TEVs to heavily decrease this threat. Furthermore, accumulation of pro-tumorigenic MDSCs also increases this inhibitory effect. TEVs have been reported to stimulate macrophage polarization towards M2 form, this ensure the TME with an increasing pool of TAM which release pro-tumorigenic growth factors, chemokines and cytokines. The consequences of TEV uptake by neutrophils is still under investigation, nevertheless their aberrant activity has been reported to be the cause of CML-like symptoms in vivo experiments. Concerning DCs, it is important to highlight the capacity of TEVs to boost the tumor immune response, transforming the communication through small EVs to a double-edge weapon against the disease.

The ability to evade the immune surveillance is one of the strategies for the generation of a proper tumor niche and a successful tumor development [80,81]. This process is not only visible in the area of the primary tumor but it is also a way to generate pre-metastatic and metastatic niches [80]. One quality of the TEVs is to carry molecules which are used in several level of communications with the surrounding cells of the microenvironment. Through receptor-mediated uptake, TEVs release their content into the cytoplasm by directly fusing with the cell membrane. Furthermore, TEV-carried ligands are recognized by proper receptors on target cells; for example, antigens carried by TEVs can bind to major histocompatibility complex (MHC) receptors [82]. Altogether, it is believed that small EVs play an important role in the communication of tumor cells to non-malignant bystander cells in their surroundings, in order to create a tumor-friendly environment (Figure 1).

### 3.1. B Cells

B cells are an essential component of the immune system. They are mainly responsible for modulating immune response and inflammation through the production of antibodies and the promotion of T cell activation and proliferation through antigen presentation [83]. Recent studies have revealed a new category of B cells, known as regulatory B cells (Bregs), involved in the control of anti-tumor immune response and tumor development [84,85,86,87,88]. Bregs possess protective functions, maintain immune tolerance and suppress pathological autoimmune and inflammatory responses [89,90,91]. Despite that, Bregs have been shown to play an important role in supporting cancer immune escape through the release of anti-inflammatory mediators, such as interleukin-10 (IL-10) [91]. Release of IL-10 allows Bregs to regulate CD4^+^ T cell differentiation, proliferation and activity (cytokine secretion), for instance by pro-apoptotic signals [90,92,93]. In a similar way, Bregs suppress CD8^+^ T cell immune response and function [94,95,96], natural killer IFN-γ production [97], monocyte proliferation and cytokine release [98] and finally dendritic cell IL-12 production [99]. Furthermore, the release of IL-10 promotes the expansion of Bregs which cause an enhanced immune depression together with IL-10 dependent stimulation and expansion of Tregs [97,100,101].

Bregs are playing an important role in lymphoma. IL-10 is rapidly produced and released by Bregs stimulated with lymphoma-derived small EVs [102]. Furthermore, small EVs released by Burkitt’s lymphoma cell lines stimulate B cell proliferation, induction of activation-induced cytidine deaminase (AID), and the production by B cells of circle and germline transcripts for IgG1 [103].

Haque and Vaiselbuh suggested that acute lymphoblastic leukemia (ALL)-derived small EVs regulate in vitro leukemic and non-leukemic B cell proliferation through the transport of proliferative, pro-survival and anti-apoptotic factors [104]. Finally, Patel et al. reported that primary human ALL-derived small EVs are released by the growing leukemia clones to promote proliferation and survival of the low density growing clones [105].

Despite having canonical protective activities, B cells and Bregs, targeted by TEVs, can be used by the tumor to circumvent the immune system and enhance tumor growth.

### 3.2. T Cells

Interaction between TEVs and T cells takes place through the interaction with surface molecules which generate signals resulting in sustained Ca^2+^ flux and activation of downstream signaling pathways, leading to alterations in the T cell transcriptome [82].

Cancer cells use TEV potential in order to diminish T cell function and thus weaken the overall immune response. Whiteside et al. reported that TEVs mediate the inhibition of CD3ζ chain expression and drastically reduce its mRNA levels [106]. Decreased expression of the T-cell receptor (TCR) ζ-chain has been reported in several autoimmune, inflammatory and malignant diseases (e.g., lymphoma), and it is commonly associated with suppression of T cell proliferation and altered cytokine production [107,108,109,110]. During cancer progression, T cells are found in close contact with tumor cells and represent an essential component of the TME. In line with this, it was shown that subverted CD4^+^ T cell subsets within the tumor microenvironment may exhibit a tumor-promoting activity [111].

Hematopoietic malignancies actively use small EVs to strike on T cells and cause a wide range of effects meant to reduce T cell actions on tumor development. Smallwood et al. demonstrated that autologous patient CD4^+^ T cells internalize chronic lymphocytic leukemia (CLL)-derived small EVs containing miR-363 that targets the immunomodulatory receptor CD69, which leads to inhibit the migration of effector T cells [112,113,114]. In another study, Diffuse Large B Cells Lymphoma (DLBCL)-derived small EVs were shown to be rapidly captured by T cells, leading to either PD-1 up-regulation or to pro-apoptotic signals, probably due to increased expression of Fas, FasL, and TRAIL [55,115].

Chemotherapy is still widely used in cancer treatment. Specific TEVs are released by tumor cells under chemotherapy as a resistance mechanism to improve cancer cell survival and strongly reduce the immune response. In line with this, it was recently shown that B lymphoma-derived small EVs enriched with CD39 and CD73 are able to hydrolyse ATP released from chemotherapy-treated cancer cells into adenosine [116] which is known to affect cancer immune response causing M2-like macrophages polarization and inhibit T cell activity and proliferation [117]. Inhibition of T cell functions is essential in the process of immune escape. In this contest, TEVs are used to inhibit T cell activation and proliferation, as well as to increase pro-apoptotic signals.

### 3.3. Dendritic Cells

Dendritic cells (DCs) are widely distributed antigen-presenting cells (APCs) which have the unique capacity to induce the activation and differentiation of naive T lymphocytes [118,119].

TEVs are used against DCs to inhibit their maturation and thus, the ability to activate effector cells. Lymphoma-derived small EVs carrying molecules such as TGF-β, IL-6 and prostaglandin E2 (PGE2) highly affect DC differentiation, maturation and function [120,121]. Stimulated by TEVs, myeloid precursors, which usually give rise to DCs in the bone marrow, differentiate as well into myeloid-derived suppressor cells (MDSCs), a subtype of myeloid cells with pro-tumorigenic and immune suppressive properties [120].

It has been shown that DCs efficiently capture DLBCL-derived small EVs, but this doesn’t cause apoptosis or upregulation of immunosuppressive mediators as it happens for T cells, but rather an enhancement of the tumor-specific immune response [122]. In according with this, a recent study suggested a potential anti-myeloma vaccine strategy using a human myeloid leukemia cell line differentiated into DCs, known as DCOne vaccine [123,124]. The potential of this cell line resides in its ability to produce a vast range of anti-MM antigens encapsulated in small EVs [125]. When co-cultured with MM patient peripheral blood, DCOne vaccine-derived small EVs boost the expansion and activation of CD8^+^ T cells. Primary MM cells co-cultured with these DCOne vaccine-activated CD8^+^ T cells were efficiently lysed [123].

The dual role of TEVs in modulating antitumor immunity is still poorly understood. Nevertheless, there is evidence for a possible modulation of the tumor immune response based on TEVs interaction with immune cells. For this reason, the attention is increasingly moving towards the use of more potent TEV-re-educated DCs to prevent, treat or eradicate tumors [126,127].

### 3.4. Natural Killer Cells

Natural killer (NK) cells represent a further component of the innate immune system. These essential cytotoxic lymphocytes control microbial infections and tumor progression in a process regulated by a balance of activating and inhibitory signals [128,129].

In cancer patients, a reduction of NK cell number as well as a depression of their activity have been previously correlated to the decrease expression of specific NK cell–activating receptors (NKp30, NKp46, NKG2C, and NKG2D) [74,130]. TEVs are able to downregulate the expression and activity of NK cell–activating receptors, among which NKG2D is the most affected. Indeed, under thermal and oxidative stress, it has been described how T- and B-leukemia/lymphoma cells release small EVs enriched in NKG2D ligands which has been suggested to act as powerful decoy to downregulate NKG2D. [74,130,131,132]. Beside this ligand-receptor interaction, also soluble growth factors released by tumor cells, and contained into TEVs, impair NK activity.

Sera of AML patients were shown to contain high levels of TEVs carrying CD33, CD34, CD117, MICA/MICB, and TGF-β1 which ultimately lead to immune suppressive effect due to decrease cytotoxic activity of NK cells. It is believed that the phosphorylation of SMAD and the down-regulation of NKG2D are the key processes to impair NK cell activity [74,106]. In line with this, CML-derived small EVs have also been found to be typically enriched in TGF-β1, which has also been shown to be essential for the tumor cell proliferation [133].

Furthermore, MM cells previously exposed to sub-lethal doses of the alkylating agent melphalan are capable of releasing small EVs stimulating the production of interferon-gamma (IFN-γ) by NK cells through a mechanism based on the activation of the nuclear factor-kappa B (NF-κB) pathway in a TLR2/heat shock protein 70 (HSP70)-dependent manner [134]. In different circumstances, MM-derived small EVs also reduce cytotoxic activity of NK cells against MM cells [135]. Furthermore, the ectoenzyme CD38, carried by MM-derived small EVs, has been suggested to convert nucleotides to adenosine, leading to an anergic immune system [136].

Finally, despite being currently under further investigation, CD38 has also been suggested as possible player of MM-derived small EV internalization in immune cells such NK, monocytes, and myeloid-derived suppressor cells, possibly leading to an alternative strategy for tumor immune escape [136]. NK cells are active players in the process of tumor cell disruption, thus TEVs are deployed with the aim to decrease NK cytotoxic activity and keep their number reduced.

### 3.5. Monocytes

Among the leukocytes, monocytes represent a subgroup of cells with a plasticity to differentiate into macrophages or dendritic cells. Monocyte plasticity is considerably reduced by TEVs. Indeed, lymphoma-derived small EVs efficiently interact with monocytes by membrane fusion, inducing secretion of the pro-inflammatory cytokines IL-6, TNF-α, IL-1β, and profoundly altering the process of their differentiation into dendritic cells [137]. In line with the previously reported changes, we showed how CLL-derived small EVs containing the non-coding RNA hY4 were able to induce monocyte polarization, leading to cytokines release, such as CCL2, CCL4 and IL-6 and expression of PD-L1, suggesting a potential small EV-based mechanism of immune escape [138].

Due to their possibility to differentiate into different immune cells, monocytes can be led to polarize into a pro-tumorigenic form, which ultimately decrease the pool of macrophages and DCs used in the fight against cancer development.

### 3.6. Macrophages

With a broad pro-inflammatory, destructive, scavenging and remodeling potential, macrophages are considered as key mediators of the immune response. Macrophages are highly plastic cells which constantly alter their functional state in response to environmental changes. The latter stimulate the expression of different surface markers and functional programs ultimately leading to the macrophage polarization [139].

The phenomenon of polarization grants macrophages a double role in cancer. Canonical activated macrophages M1 are crucial for tumor immune response due to their ability to produce pro-inflammatory cytokines and reactive oxygen/nitrogen species. The M2 variation, on the other hand, produces anti-inflammatory cytokines which not only causes a considerable reduction of tumor immune response activity but also enhancing tumor progression through angiogenesis and promotion of matrix remodeling [139].

In a cancer microenvironment, M2 macrophages are educated into Tumor-Associated Macrophages (TAMs). This category of macrophages is known to release pro-tumorigenic growth factors, chemokines and cytokines which will support tumor progression [140,141,142,143].

Macrophages behavior can be regulated through TEVs. In a subcategory of DLBCL, the innate immune-signaling adaptor myeloid differentiation primary response 88 (MyD88) has been detected in the DLBCL-derived small EVs. Manček-Keber et al. described that MyD88 is transferred into macrophages triggering the activation of pro-inflammatory signals (such as NF-κB) independent from the TLR and IL-1R receptors [144]. In another study, Chronic Myeloid Leukemia (CML)-derived small EVs have been shown to induce M2-like macrophage polarization leading to IL-10 and TNF-α overexpression. Furthermore, the downregulation of the inducible nitric oxide synthase (iNOS) causes reduction of nitric oxide (NO) and ROS levels in the TEV-treated macrophages [145]. Similarly to the monocytes, macrophages are modified by TEVs and turn against the microenvironment. Together with the B cells, TAMs are used to reshape the microenvironment by enhancing tumor immune escape and promoting angiogenesis.

### 3.7. Granulocytes

Neutrophils are the body’s first line of defense against foreign invaders and thus one of the most important key mediators of the innate immune response [146]. Neutrophils are potent antitumor effector cells due to their cytotoxic activities and ability to release cytokines and chemokines which leads to the recruitment of other cells with antitumor activity [147,148,149]. Despite this, similarly to macrophages and monocytes, neutrophils also show a phenotypic plasticity, which is modulated by different tumor-derived signals, and then display pro- or anti-tumor effects [150,151].

CML can be driven by the formation of the hybrid gene BCR/ABL kinase as results of the Philadelphia chromosome rearrangements. CML-derived small EVs have been documented to carry such gene and transfer it in vitro and in vivo to neutrophils, ultimately causing an aberrant gene expression program in the target cells, and recapitulating CML-like symptoms in Sprague-Dawley (SD) rats or NOD/SCID mice [152].

It is worth mentioning that Hansen and colleagues described that Hodgkin lymphoma (HL)-derived CD30^+^ small EVs induce the release of the pro-inflammatory cytokine IL-8 by healthy eosinophil-like EoL-1 cells and primary granulocytes [153].

Although further investigation is required, the typical antitumor activity of neutrophils appears to be easily subverted to a pro-tumorigenic one, by decreasing their cytotoxic ability and potentially increasing their involvement in inflammation.

### 3.8. Myeloid-Derived Suppressor Cells

Myeloid-derived suppressor cells (MDSCs) are a heterogeneous population of myeloid cells initially reported to hamper immune responses during chronic infections [154]. In cancer, these expanded myeloid cells contribute to tumor progression, immune evasion, and provide support to stroma [155,156,157]. Expansion of MDSCs is strongly depending on the ability of the tumor to secrete myeloid-influencing factors, such as IL-6, vascular endothelial growth factor (VEGF), PGE2 and granulocyte-macrophage colony stimulating factor (GM-CSF) [157,158,159,160].

Purified MDSCs were shown to inhibit both CD4^+^ and CD8^+^ T cell responses in vitro [161,162]. Further studies demonstrated that MDSCs down-regulate T cell functions and promote tumor metastasis by secreting a wide array of chemokines [162,163]. It has been suggested that the suppressive functions of MDSCs could have been promoted by HSP72 expressed at the surface of lymphoma-derived small EVs which activate STAT3 pathway and stimulate the production of IL-6 in a TLR2/MyD88-dependent manner [137].

Wang et al. showed that bone marrow stromal cells (BMSCs) from the MM microenvironment are able to release small EVs which are taken up by MM cells and MDSCs. In this context, BMSC-derived small EVs directly support the survival of MDSCs through a greater activation of STAT1 and STAT3 pathways in vitro. Indeed, activated MM-MDSCs acquire an enhanced T cell suppression activity in vivo which facilitates immune escape of the MM cells [164].

MDSCs are one of the most powerful population of cells involved in the siege of the microenvironment. Due to the strong pro-tumorigenic potential of MDSC, tumor cells proliferate undisturbed thanks to the effective immune evasion and nutrient support.

An overview of the hematological malignancy-derived small EV functions based on the content and immune cells is summarized in Table 1.

## 4. Hematological Malignancies-Derived Small EVs and Stromal Cells

**Figure 2:**
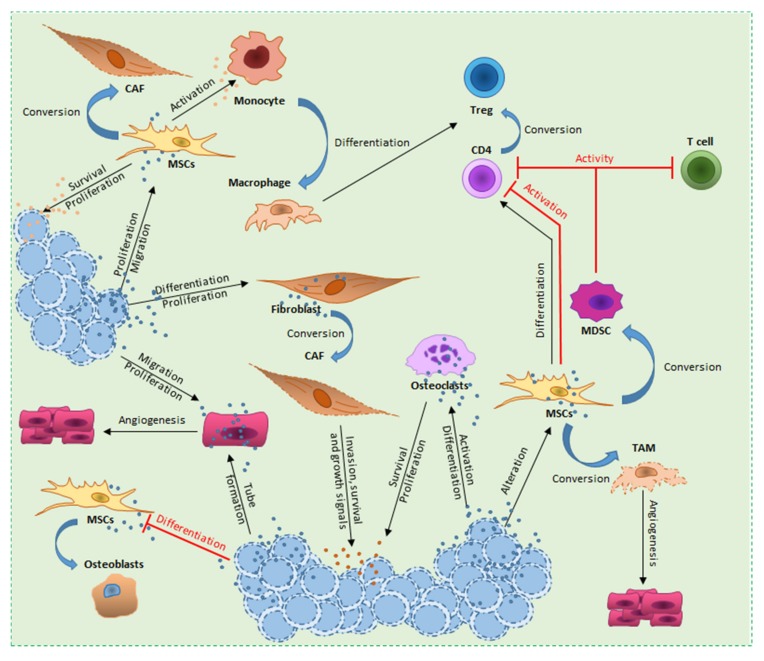
Overview of hematological malignancy-derived small EV effects on stromal cells. In the tumor-stroma communication, TEVs play an essential role in re-shaping the microenvironment. Small EVs coming from different tumors have been described to induce differentiation and increase proliferation of fibroblasts. Through the transfer of different molecules, TEVs convert fibroblasts and MSCs into CAFs which in turn boost tumor invasion, survival and growth. TEVs have the ability to block the MSCs physiological differentiation into osteoblasts causing a progressive bone loss. Under the activity of TEVs, MSCs are further altered in proliferation and migration, resulting in the release of MSC-derived small EVs which in turn boost survival and proliferation of the tumor cells. MSC-derived small EVs mediate immunosuppression in various immune-effector cells, with Tregs being one of the most deregulated immune cell subtype. Indeed, MSC-derived small EVs directly target CD4^+^ T cells, thus reducing their activation and expansion towards Treg compartment. Furthermore, the aberrant activation of monocytes leads to differentiation into macrophages and consequently Tregs expansion. ECs are an essential component of the microenvironment necessarily for angiogenesis. ECs are actively targeted by TEVs with the aim to increase vascularization in the tumor surrounding, leading to higher proliferation and tumor spreading. Finally, osteoclasts, a further component needed for bone remodeling, under the effect of TEVs show a deleterious activation and differentiation which lead to bone loss and pro-tumorigenic chemokine and growth factor release.

The stroma consists in heterogeneous cell populations delivering structural and physiological support for hematopoietic cells. In cancer, the stroma evolves in parallel to the disease and it is considered as one of the essential key factors for tumor development, progression and dissemination [165,166,167,168] (Figure 2).

### 4.1. Fibroblasts

Fibroblasts are extremely versatile and resilient cells distributed in different parenchymal tissues. They represent the main source of extracellular matrix (ECM) proteins which provide a scaffold for cells but also strongly influence their cell phenotype and functions [169]. Furthermore, fibroblasts also function as accessory cells in many immune and inflammatory responses [170].

Cancer-associated fibroblasts (CAFs) are one of the most dominant cell types found in solid tumor lesions and play a key role in the tumor niche, producing cytokines, chemokines, metabolites, enzymes and extracellular matrix molecules which will ultimately fuel cancer cells [169,171]. Moreover, CAFs regulate the inflammatory microenvironment by expressing pro-inflammatory genes, such as IL-1β, IL-6, IL-8, TGF-β, CXCL12, and collagen [172,173,174,175].

The impact of hematopoietic malignancy-derived small EVs on fibroblast behavior has been reported in multiple studies. CML-derived small EVs has been described, both in vitro and in vivo, to be able to induce fibroblast-like bone marrow stromal cells to release IL-8, ultimately supporting CML cells survival [176]. AML-derived small EVs have been described to transport key mRNAs, such as CXCR4 and IGF-IR (insulin-like growth factor-I receptor), directly to fibroblasts [177]. Huan and colleagues showed that these TEVs modulate fibroblasts proliferation as well as promote VEGF expression in vitro, thus altering angiogenesis responses [177]. Frassanito and colleagues also demonstrated that myeloma-derived small EVs are able to increase myeloma-fibroblasts proliferation and survival via the transfer of miR-27b-3p and miR-214-3p [178]. T-cell leukemia- and CML-derived small EVs have been described to contain the telomerase (hTERT) enzyme mRNA and the potential transfer to hTERT-negative fibroblasts [179] may prime to aberrant hTERT expression in TEVs-targeted fibroblasts potentially leading to a tumor-like phenotype [180,181].

Fibroblasts are an important component of every tissue. The transformation into CAFs causes deep changes in the microenvironment architecture, causing an increase in inflammation which, together with small EVs communication, leads to tumor advance.

### 4.2. Mesenchymal Stem Cells

Mesenchymal stem cells (MSCs) origin from the stromal compartment and present properties related with stem cells. Actually, MSCs are adult multipotent cells capable of self-renewal and of differentiating into several cell types, e.g., osteoblasts, chondrocytes and adipocytes [182].

MSCs interaction with cancer cells results in dramatic changes in the MSCs phenotype and functions. Indeed, MSCs present in the TME transdifferentiate into M2 macrophages and myeloid-derived suppressor cells (MDSC) under the influence of cytokines or chemokines [183,184].

MM-derived small EVs carrying high levels of miR-21 and miR-146a significantly increase MSCs proliferation. [185]. Adult T-cell leukemia/lymphoma (ATLL)-derived small EVs are deployed for transfer epigenetic mediators, such as miR-21 and miR-155, which ultimately promote proliferation of human MSCs [186]. We demonstrated in vitro that interaction of MSCs with CLL-derived small EVs causes a shift towards a CAF-like phenotype characterized by expression of CXCL12, α-smooth muscle actin (α-SMA) and fibroblast surface protein (FSP) [187]. Furthermore, CAFs support the niche promoting CLL cell migration/invasion (ICAM1 and MMP1), survival (c-IAP2) and growth in vivo [187]. Finally, AML-derived small EVs carrying miR-7977 have been described to reduce MSCs ability to support CD34^+^ progenitor cells in the bone marrow, resulting in alterations in proliferation and migration MSCs and hematopoietic progenitor [188].

In order to maintain a favorable microenvironment, CLL cells were demonstrated to release small EVs which has the potential to sustained the activation of the AKT, mTOR, p70S6K and HIF-1α pathways, furthermore they modulate the AKT-GSK3β or AKT-β-catenin signaling pathways [189]. Similar findings were reported with MM-, ALL- and AML-derived EVs creating a pre-metastatic niche by activating signaling cascades in the BM-MSC; the remodeling of the surrounding bystander stromal cells ultimately leading to drug resistance, cancer progression, dissemination and metastasis [190,191].

It has been shown that, with the aim to promote in vitro and in vivo survival, CML cells release small EVs which stimulate MSCs to produce IL-8 [176]. Furthermore, Corrado and colleagues showed that CML-derived small EVs are enriched in amphiregulin (AREG), which cause aberrant activation of EGFR in MSCs, ultimately leading to increased expression of SNAIL and its targets, such as MMP9 and IL8 [192].

MSC-derived small EVs mediated pro-tumorigenic effects preserve the cross-talk with the cells from the TME, including tumor cells. In line with this, MSCs participate in the re-education of the microenvironment providing a framework for anchoring tumor cells and secreting multiple bioactive factors which directly alter the neighboring cells for what concern survival, apoptosis, maturation and differentiation [193,194,195]. Under the effect of MM cells and MM-derived small EVs, MSCs release pro-tumorigenic small EVs which ultimately stimulate the disease progression. Indeed, MSC-derived small EVs transfer miR-15a to MM cells improving survival and proliferation [196]. The same group also demonstrated that treatment of MSCs in vitro with CML-derived small EVs promotes leukemia cells adhesion to the stromal cells supporting continuous growth and invasiveness of CML cells [192].

MSC-derived small EVs have been described to mediate immunosuppression in various immune-effector cells [197,198,199]. In line with this, MSC-derived small EVs carry CD39 and CD73 ectonucleotidases, which catalyze adenosine production, and immunosuppressive factors, including indoleamine 2,3-dioxygenase (IDO), TGF-β, IL-6, PGE2, PD-1, galectin-1 and HLA-G5 [200,201,202,203].

T cells treated in vitro with MSC-derived small EVs show a reduction in activation and an expansion towards the Treg compartment [204]. MSC-derived small EVs also activate monocytes by the TLR signaling pathway [197,204]. By consequence, monocytes differentiate into macrophages which secrete IL-10, leading to Tregs expansion and enhancing the effects induced by MSCs [197,205].

As a major component of the TME, MSCs play a key role in promoting tumor progression. Indeed, on one hand TEVs are used to enhance MSCs proliferation, survival and promote pro-tumorigenic phenotype. On the other hand, MSCs guarantee suppression of the immune system, pro-survival signals and induction of polarization for different immune cells.

### 4.3. Endothelial Cells

Endothelial cells (ECs) are the active players in the formation of new blood vessels both in health and diseases. In cancer, angiogenesis is an essential process that allows tumor cells to proliferate, spread and survive [206,207].

Several studies have suggested a strong impact of TEVs on the regulation of EC proliferation and migration, making them an active tool to be added to the already known cancer pro-angiogenesis factors (e.g., VEGF). AML-derived small EVs are enriched in pro-angiogenesis transcripts, VEGF and VEGF receptor (VEGFR), which have been demonstrated to be transferred into ECs leading to vascular remodeling by increasing EC glycolysis [208]. MM pathogenesis is deeply dependent on modification of the bone marrow microenvironment. In order to achieve this, MM releases small EVs carrying multiple angiogenesis-inducing proteins, such as STAT3, JNK1/2/3, ERK1/2 and p53, thus actively promoting EC migration, proliferation and resistance to apoptosis [208,209,210].

Indeed, pre-treatment of MM cells with bortezomib, a known proteasome inhibitor, led to the release of TEVs characterized by major differences in content, size and possessing anti-angiogenesis properties [209]. Analogously, Taverna and colleagues showed that treatment of ECs with CML-derived small EVs led to an increase in IL-8 and VCAM1 levels, ultimately enhancing angiogenesis. On the other hand, CML cells treated with curcumin release TEVs containing miR-21 which decrease EC migration and proliferation [211,212].

We have also shown that the uptake of CLL-derived small EVs by the ECs causes the up-regulation of CXCL1, IL34 and ICAM1 genes in targeted ECs [187]. Furthermore, TEV uptake led to increase angiogenesis ex vivo and in vivo boosting leukemia cells proliferation [187]. In a further study, Umezu and colleagues shown that CML-derived small EVs are enriched in miR-17-92 cluster. Deeper analysis demonstrated that miR-92a transferred into ECs confers pro-angiogenic effects, leading to endothelial migration and angiotube formation [213]. Concerning CML, miR-126 has been identified as component of the LAMA84 human cell line (CML at blast crisis)-derived small EVs [214]. Here, the author suggested a singular phenomenon in which TEVs are not directly involved in triggering angiogenesis, but rather used by CML cells to detach from EC monolayer, possibly through decrease of VCAM1 transcript, and migrate towards a richer source of nutrients [214].

Finally, a recent study revealed that Piwi-interacting RNAs (piRNAs), known to be important regulators involved in the MM pathogenesis, are found in the same tumor small EVs [215]. In particular, MM-derived small EVs enriched in piRNA-823 boost proliferation, tube formation and invasion of the targeted ECs [215].

Consistent with the current knowledge of hypoxia being a key driver of angiogenesis, under in vitro chronic hypoxic conditions, MM cells have been described to release high amount of small EVs enriched in miR-135b [216,217]. Transferred miR-135b leads to the direct suppression of the factor-inhibiting hypoxia-inducible factor 1 (FIH-1) in ECs, enhancing endothelial tube formation via the HIF signaling pathway [217]. Similarly, Tadokoro and colleagues have described that under hypoxic condition CML-derived small EVs contain a singular subset of miRNAs, including miR-210, which significantly enhanced tube formation in tumor targeted ECs [218]. Finally, a study performed on a subtype of AML, Acute Promyelocytic Leukemia (APML; AML-M3) expressing the oncogenic fusion protein PML-RARα, detected the PML-RARα transcript in TEVs [219]. ECs targeted by these particular TEVs result in more tissue factor-positive and pro-coagulant cells. This effect is highly decreased if the APML cells are treated with all-trans retinoic acid (ATRA), a PML-RARα antagonist typically used in the clinic [219].

Cancer proliferation, survival and metastasis dissemination are highly dependent on angiogenesis. To this end, TEVs are used to boost ECs proliferation, migration and tube formation.

### 4.4. Osteoblasts and Osteoclasts

Bone is a complex and dynamic tissue that requires the collaboration of different group of cells (e.g., osteoblasts, osteoclasts and ECs) in order to establish new bone formation and guarantee the tissue maintenance, repair and remodeling [220,221]. Disruption of any of the components taking part on these processes causes bone pathologies and contributes to tumor development [222,223].

Differentiated MSCs give rise to osteoblasts which are a class of cells actively involved in bone formation [220,224,225]. Severe osteoblastic defects have been correlated, both in xerograph and transgenic mouse models, with CML and AML [226,227,228]. On the other hand, presence of functional active osteoblasts has been described to reduce tumor size and increase animal overall survival [229]. Kumar and colleagues have described how AML-derived small EVs suppress normal hematopoiesis, causing decrease in osteogenesis [230]. The study further describes that the loss of the osteoblast population is principally due to alterations driven by AML-derived small EVs to mesenchymal progenitor differentiation potential [230].

Osteoclasts are specialized cells, originating by the fusion of multiple precursor cells (characteristic multinuclear phenotype), involved in bone break-down and reshaping [220,221]. One of the features of MM is the progressive disruption of the bone tissue leading to osteolytic diseases. This goal is reached by increase osteoclastic differentiation and activity in parallel with osteoblastic suppression activity [231]. Osteoclast activation leads to cytokine and growth factor release and causes progressive bone reduction [232,233,234]. In a recent study, Raimondo and colleagues identified amphiregulin (AREG) as a specific cargo molecule in MM small EVs [235]. AREG enriched MM-derived small EVs have been shown in vitro to boost pre-osteoclast differentiation stimulating EGFR pathway. Furthermore, MSCs in contact with AREG enriched MM-derived small EVs shown a differentiation block towards osteoblasts [235]. A similar study on Raw264.7, a murine pre-osteoclastic cells line, described how small EVs from MM cell lines and primary MM patients induce osteoclast differentiation by increasing CXCR4 expression and pro-survival pathways [236].

In another in vitro model, Faict et al. further proved the ability of MM-derived small EVs to impact on osteoblast function and differentiation via transferring of Dickkopf-related protein 1 (DKK-1) resulting in Runx2, Osterix, and Collagen 1A1 downregulation [237]. In the same study, the administration of MM-derived small EVs caused important osteolysis in vivo [237].

Reshaping the surrounding microenvironment is an essential feature requested for hematological malignancies in order to develop, progress and disseminate [238]. Indeed, to generate an appropriate bone niche is necessary to escape the immune system, improve chemoresistance, increase nutrients uptake and metastatic potential.

An overview of the hematological malignancy-derived small EV functions based on the content and stromal cells is summarized in Table 2.

## 5. Conclusions

Cancer is a complex disease which doesn’t involve only tumor cells but also a composite cellular microenvironment. Through the multiple strategies and tools deployed by cancer cells to gain proliferative and survival advantages, small extracellular vesicles are one of the most concealed. These vesicles are commonly released by all cells and they are typically used by the cells to communicate with each other. In cancer, small EVs are used to overload the surrounding tissues with pro-tumorigenic signals, derived from TEVs but also from microenvironment cells modified by TEVs.

In this review, we presented how hematological malignancy-derived small EVs possess extremely high potential to re-educate normal tissues, and thus, to re-shape the surrounding tumor microenvironment.

In an initial hostile microenvironment, tumor cells need to alter the normal tissue cell composition to establish a proper niche which will be necessary for cancer growth. Immune cells are the first line of defense against aberrant cells escaped from molecular regulators. Hematological malignancy-derived small EVs actively hijack the immune system guaranteeing a more rapid and successful cancer development. Immune effector cells possess the ability to eradicate cancer cells, thus TEVs are used with the aim to eliminate such threat reducing function, proliferation and migration of effector cells. Hematological malignancies, such as lymphomas and CML, directly target NK cells with small EVs containing molecules which reduce or completely block the cytotoxicity [130,131,133]. A similar strategy is used by DLBCL-EVs to directly regulate immune checkpoint receptor expression or induce apoptosis in T effector cells [55,115].

Cell polarization is another process driven by TEVs to mine the natural immune functions. Through polarization, TEVs change the behavior of certain highly plastic cells, such as monocytes and macrophages, making them gain specific pro-tumorigenic phenotype and function. Under CLL small EVs, monocytes are subjected to polarization that causes changes in immune checkpoint composition, leading them to block T cells activity, and release of pro-inflammatory cytokines [138]. The latter is also induced by macrophages upon CML-derived small EVs uptake [144]. Inflammation has an essential effect in the tumorigenesis as it co-participates in reshaping the microenvironment, supporting tumor growth and favoring gene instability. To guarantee a local degree of inflammation is a key feature of cancer and is also an essential process necessary to establish and maintain pre-metastatic niches.

An effective strategy to enhance the bypass of the immune defenses is to hit also regulatory cells which aim to maintain effector cells aware and active. Through the use of small EVs, T lymphoma blocks the maturation of essential patrolling cells such as DCs [121] making them incapable to stimulate T cell and inducing their differentiation into MDSCs. MM-derived small EVs, in the other hand, directly target MDSCs leading to their expansion and a switch towards pro-tumorigenic phenotype [164]. Rather than decrease MDSCs activity, MM uses MDSCs immune regulatory ability to inhibit functions of essential effector cell such as CD4^+^ and CD8^+^ and NK cells.

Essential for a properly regulated immune response, Bregs and Tregs are used by the tumor to enhance the immune suppression of an already lowered immune system. Lymphoma-derived small EVs were described to cause a persistent activation and expansion of Breg via increased release of IL-10, this causes a deep depression in function and proliferation of effector cells, together with expansion of Tregs [97,98,102,103].

Deregulation of both effector and regulatory immune cells through the use of small EVs is an elegant and efficient strategy to hijack immune defenses and allow cancer progression.

Stroma composition evolves in parallel to cancer growth and progression. Indeed, communication with stromal cells allows to generate an appropriate niche surrounding the primary tumor, and potentially in secondary organs, to proliferate and welcome metastatic cells, respectively.

During hematological malignancy development and progression, bones lose the structural protection typically attributed to this tissue. During these processes there is a progressive increase in osteoclasts and decrease in osteoblasts, which break down and produce new bone, respectively. MM is known to continuously modify the cellular component and structure of bone. MM-derived small EVs have been described to transfer molecules responsible for pre-osteoclast rapid differentiation leading to osteoclast expansion and activation [232,233,234,235]. In turn, osteoclasts release high amount of cytokines and growth factors which ultimately increase cancer cell proliferation and survival [232,234]. The increase in osteoclasts causes a progressive bone loss which is further enhanced by the decreased amount of osteoblasts. In line with this, MM-derived small EVs impact on osteoblast function and differentiation, overall leading to osteolysis and a more rapid dissemination of MM cells in the organism [237]. Bone reshaping by MM-derived small EV is not exclusively given by a direct transfer of molecules into the osteoblasts, but also by reducing MSC differentiation potential [235].

Due to their stem cell like properties, MSCs can differentiate in various cell types. This property is used by tumor cells to increase immune response depression, local and distant inflammation and increase tumor pro-survival, proliferation and invasion signals. As previously mentioned, MSCs have a strong impact on immune suppression. Depending on the hematological malignancy, small EVs possess specific molecules which cause distinct effects on MSCs. For instance, lymphoma- and MM-derived small EVs induce MSCs to highly support MDSC population expansion [161,162,164]. In the other hand, MSCs targeted by CLL-derived small EVs are characterized by different gene expression and phenotypical changes. Similarly to fibroblasts, CLL-derived small EVs lead MSCs to differentiate into CAFs, causing release of pro-inflammatory cytokines in the TME, together with pro-survival, growth and migratory signals towards the cancer cells [187]. Once activated by TEVs, MSCs can, in return, release small EVs directed to immune cells to enrich the pool of pro-tumorigenic immune cells and decrease the anti-tumor activity of effector cells [197,204].

In line with this, monocytes activated by MSC-derived small EVs rapidly differentiate in macrophages, increasing the pool of TAM in the microenvironment. Accumulation of TAMs from MSCs is also correlated with an increase in angiogenesis [139]. The formation of new blood vessels, allowing nutrients and oxygen uptake, is another essential process required for tumor development and, in advances stages, tumor cells dissemination. Different hematological malignancy-derived small EVs contain various pro-angiogenic factors which trigger ECs proliferation, survival and tube formation [208,213,217].

Through the release of small EVs, tumor cells can interact with an impressive number of cells, tissues and structures. It is important to stress how the understanding of small EV role during tumor development and progression is essential in order to develop effective anticancer strategies. Achieving a deeper knowledge of this intricate communication system would allow us to identify its weaknesses, as well as to use it as a potential drug delivery strategy.

## Figures and Tables

**Table 1:** Overview of the hematological malignancy-derived small EV functions based on the content and immune cell types.

Hematopoietic Malignancies	Small EVs Content	Target Cells	Effects	References
Lymphoma	LMP1	B cells	Increased proliferation	[103]
CLL	miR-363	T cells	Migration inhibition	[114]
DLBCL	Fas, FasL, and TRAIL	T cells	Increased apoptosis	[55,115]
Lymphoma	CD39 and CD73	T cells Macrophages	Accumulation of Adenosine	[116,117]
T Lymphoma	TGF-β, IL-6 and PGE2	DCs	Block of maturation	[121]
T- and B-leukemia/lymphoma	NKG2D ligands and TGF-β	NK cells	Reduced cytotoxicity	[130,131]
CML	TGF-β	NK cells	Reduced cytotoxicity	[133]
AML	CD33, CD34, CD117, MICA/MICB, and TGF-β	NK cells	Reduced cytotoxicity	[74,106]
MM	HSP70	NK cells	Activation of the NF-κB pathway and IFN-γ released	[134]
CLL	Y RNA hY4	Monocytes	Polarization induction	[138]
DLBCL	MyD88	Macrophages	Pro-inflammatory signals	[144]
CML	BCR/ABL	Neutrophils	Aberrant BCR/ABL expression and CML-like symptoms	[152]
Lymphoma	HSP72	MDSCs	Suppressive functions	[137]
MM	HSP72	MDSCs	Proliferation increased and activity enhanced	[164]

Disease abbreviations: Chronic Lymphocytic Leukemia (CLL); Diffuse Large B Cell Lymphoma (DLBCL); Chronic Myeloid Leukemia (CML); Acute Myelogenous Leukemia (AML); Multiple Myeloma (MM).

**Table 2:** Overview of the hematological malignancy-derived small EV functions based on the content and stromal cell types.

Hematopoietic Malignancies	Small EVs Content	Target Cells	Effects	References
AML	CXCR4 and IGF-IR	Fibroblasts	Proliferation and VEGF expression	[177]
MM	miR-27b-3p and miR-214-3p	Fibroblasts	Myeloma-fibroblasts proliferation and survival boost	[178]
T cells Leukemia and CML	hTERT	Fibroblasts	CAF phenotype acquisition	[179,180,181]
CLL	microRNA and Y RNA	MSCs	CAF phenotype acquisition	[187]
MM	miR-21 and miR-146a	MSCs	Proliferation and CAF induction	[185]
ATLL	miR-21 and miR-155	MSCs	MSCs proliferation	[186]
AML	miR-7977	MSCs	Reduced ability to support CD34^+^ cells in the bone marrow	[177]
CML	AREG	MSCs	Aberrant activating of EGFR signalling	[192]
MM	AREG	MSCs	Block differentiation towards osteoblasts	[235]
AML	VEGF and VEGFR mRNA	ECs	Pro-angiogenesis mediated by increased glycolysis	[208]
MM	STAT3, JNK1/2/3, ERK1/2 and P53	ECs	Enhanced migration and tube formation	[209,210]
MM	miR-135b	ECs	Aberrant HIF-FIH signalling	[217]
CML	miR-210	ECs	Enhanced tube formation	[218]
CML	miR-17 -92 cluster	ECs	Enhanced migration and tube formation	[213]
MM	piRNA-823	ECs	Enhanced proliferation, tube formation and invasion	[215]
CML (Blast crisis)	mir-126	ECs	Increased tumor cells migration	[214]
APML; AML-M3	PML-RARα mRNA	ECs	Acquisition of pro-coagulant and tissue factor properties	[219]
MM	AREG	Pre-osteoclasts	Increased differentiation towards osteoclasts	[235]
MM	DKK-1	Osteoblasts	Block of function and differentiation	[237]

Disease abbreviations: Chronic Lymphocytic Leukemia (CLL); Chronic Myeloid Leukemia (CML); Acute Myelogenous Leukemia (AML); Multiple Myeloma (MM); Adult T-cell Leukemia/Lymphoma (ATLL); Acute Promyelocytic Leukemia (APML).
